# Bob Sim, Memories from Oxford and Grenoble

**DOI:** 10.3390/v13040561

**Published:** 2021-03-26

**Authors:** Gérard J. Arlaud

**Affiliations:** Commissariat à l’Energie Atomique et aux Energies Alternatives, Direction des Sciences du Vivant, Institut de Biologie Structurale, F-38027 Grenoble, France; arlaud.gerard@gmail.com

I initially met Bob and Edith Sim when they moved to Grenoble in 1976 for a two-year fellowship at the Centre d’Etudes Nucléaires. Edith was working in the same Department but in a different lab next door, whereas Bob and I were both working in the Laboratoire d’Immunochimie under the supervision of Maurice Colomb. Bob and I shared a common interest for C1, the triggering complex of the classical pathway of complement, a molecule that was quite poorly understood at that time. Together we published a series of articles dealing mainly with methods for the purification of C1 sub-components and C1-inhibitor, and with the inhibition of the C1r and C1s proteases of C1, showing that C1-inhibitor not only blocked their activities but also completely dissociated the C1 complex. This period was scientifically very fruitful, and Bob’s contribution in terms of ideas and methods was very stimulating.

In my turn, I left Grenoble in 1980–1981 with my family and went to Oxford for a post-doctoral fellowship in the MRC Immunochemistry Unit of the Biochemistry Department, and this gave me the opportunity to join Bob and Edith, who were back at Oxford after their French stay. They greatly facilitated our move to Oxford by finding us a nice house in Summertown, a pleasant area in the northern part of the city. Bob and Edith also kindly gave us all sorts of practical advice about the day-to-day Oxford way of life, including, for example, the best shops in the covered market, where to buy good French baguettes, or where to watch good movies. My scientific goal was to decipher the amino acid sequence of C1r, and I was mainly collaborating for this purpose with Jean Gagnon, but I was working in the same lab as Bob and Edith, and enjoyed every day their company and advice. At that time, a number of members of the MRC Immunochemistry Unit, including Bob and Edith, Ken Reid and Duncan Campbell were from Scotland, and the whole lab was ritually invited at Ken’s place each year for Robert Burns Day to eat haggis and drink whisky. I still remember these very special moments. I also have excellent memories of the friendly atmosphere in the lab and outside, including the traditional gathering at a pub nearby each Friday after work. I am indebted to the members of the Immunochemistry Unit, and particularly to Bob and Edith for this enriching and fruitful experience.

Gerard Arlaud
Institut de Biologie Structurale, Grenoble
(retired 2010)

